# Clinical Holistic Medicine: The Case Story of Anna. III. Rehabilitation of Philosophy of Life During Holistic Existential Therapy for Childhood Sexual Abuse

**DOI:** 10.1100/tsw.2006.338

**Published:** 2006-03-07

**Authors:** Søren Ventegodt, Birgitte Clausen, Joav Merrick

**Affiliations:** ^1^ Nordic School of Holistic Medicine, Teglgårdstræde 4-8, DK-1452 Copenhagen K, Denmark; ^2^ Quality of Life Research Clinic, Teglgårdstræde 4-8, DK-1452 Copenhagen K, Denmark; ^3^ Quality of Life Research Center, Teglgårdstræde 4-8, DK-1452 Copenhagen K, Denmark; ^4^ Scandinavian Foundation for Holistic Medicine, Sandvika, Norway; ^5^ Vejlby Lokalcenter, Vejlby, Denmark; ^6^ National Institute of Child Health and Human Development, Ben Gurion University of the Negev, Beer-Sheva, Israel; ^7^ Center for Multidisciplinary Research in Aging, Ben Gurion University of the Negev, Beer-Sheva, Israel; ^8^ Zusman Child Development Center, Ben Gurion University of the Negev, Beer-Sheva, Israel; ^9^ Faculty of Health Sciences, Ben Gurion University of the Negev, Beer-Sheva, Israel; ^10^ Office of the Medical Director, Division for Mental Retardation, Ministry of Social Affairs, Jerusalem, Israel

**Keywords:** quality of life, QOL, philosophy, human development, holistic medicine, public health, holistic health, holistic process theory, life mission theory, group therapy, incest, violent abuse, rape, sexual torture, existential healing, existential (Antonovsky) coherence, Denmark

## Abstract

When we experience life events with overwhelming emotional pain, we can escape this pain by making decisions (in our mind) that transfer responsibility from our existence to the surrounding world. By doing this, we slowly destroy the essence of our being, health, quality of life, and ability to function. The case of Anna is an excellent example of such a systematic destruction of self, done to survive the extreme pressure from childhood abuse and sexual abuse. The case study shows that the damage done to us by traumatic events is not on our body or soul, but rather our philosophy of life. The important consequence is that we can heal our existence by letting go of the negative decisions taken in the past painful and traumatic situations. By letting go of the life-denying sentences, we come back to life and take responsibility for our own life and existence. The healing of Annas existence was done by existential holistic therapy. Although the processing did not always run smoothly, as she projected very charged material on the therapists on several occasions, the process resulted in full health and a good quality of life due to her own will to recover and heal completely. The case illustrates the inner logic and complexity of intensive holistic therapy at the most difficult moment, where only a combination of intensive medical, psychiatric, and sexological treatment could set her free. In the paper, we also present a meta-perspective on intensive holistic therapy and its most characteristic phases.

## INTRODUCTION

In order to find a way to rehabilitate victims of childhood sexual maltreatment, many forms of therapy have been used[1,2,3,4,5,6,7,8,9,10,11,12,13,14], but not always with satisfactory results. Therefore, we need to develop new methods and therapeutic tools to facilitate the important process of existential, emotional, mental, and sexual healing that can take the patient all the way back to a normal life. We have seen such a treatment with one of our patients and believe it is important to analyze and reflect over her journey through a long treatment process.

Anna was a borderline patient[15,16,17], student, 22 years of age, who had completely repressed over 100 episodes of childhood sexual abuse. She has recovered completely, including regaining her full emotional range, through holistic existential therapy[18], individually and in a group[19,20]. The therapy took 18 months and more than 100 hours of intensive holistic existential therapy. In the beginning of the therapy, the issues was her physical and mental health[21,22]; in the middle of the therapy, the central issue was about her purpose of life[23] and her love life; and at the end of the therapy, the issue was about gender, character, spirit, and sexuality. The strategy was to build up her strength for several months, mobilizing all her hidden resources and motivation for living, before the painful old traumas were confronted and integrated. The therapy was based on the quality of life philosophy[24,25,26,27,28,29, 30,31] and theoretically based on the life mission theory[23], the theory of ego[32], the theory of talent[33], the theory of the evil side of man[34], the theory of human character[35], and the holistic process theory of healing[18]. The clinical procedures included conversational therapy and training in philosophy of life[36]. The tools in use were the advanced holistic medical toolbox and the group therapeutic tools[19], extended use of therapeutic touch[37], holistic pelvic examination[38,39], acceptance through touch[40], and acupressure though the vagina[41] in order to integrate the early traumas bound to the pelvis and scar tissue in the sexual organs.

The therapy had two phases; the first was a normal phase, where the patient was integrating old material destabilizing her mental state[21,22]. After months of therapy, she broke through to a layer of repressed material revealing substantial sexual abuse. The traumas started as physical replay of rape traumas followed by the associated emotions and feelings, and finally came her insight and understanding, leading her to identify and let go of hundreds of negative sentences, the content of which is the issue of this paper.

It seems as if she worked her way up the scale of existential responsibility, from the hallucinated state in the bottom to the free and responsible state at the top of the scale (see Table 1)[22]. The scale describes how existential responsibility — seen from inside (the state of consciousness) and outside (the behavior) — is first lost and then found as the patient climbs the ladder of hallucination, blacking out, denial, escape, psychic death, unbearable emotional pain, to freedom of perception. To rehabilitate a psychotic patient in a hallucinatory state of consciousness, you need to help him or her confront the trauma that originally motivated the escape into hallucination. In doing this, you must carefully avoid pushing them deeper down into suicide[22].

Just before the end of the therapy, Anna had a severe existential crisis, where she confronted the value of her own life and she decided to live and accept life as it is, including the shadow of herself and the experiences of evil in her personal history. Interestingly, when she was healed at the end of therapy, she had to go to the bottom of the scale to confront death with her totality, to finally win life and assume full responsibility and her freedom, which was lost in the past.

## THE NEGATIVE, LIFE-DENYING DECISIONS

Table 2 is a list of the most important negative and life-denying sentences that were released during the therapy. The sentences were the essence of the gestalts that were integrated in the therapy; they are both feelings and thoughts at the same time, making them extremely *to the point* of the experience. In Table 3, the sentences are listed according to the responsibility for life scale.

**TABLE 1**
**Responsibility for Life Scale**

| Degree of Responsibility for Your Own Existence (Estimated Percentage) | State of Consciousness (Many Substates Exist) | Behavior (Other Patterns Might Exist) |
|---|---|---|
| 100% responsibility **Mentally healthy** | Present, fully aware, interpreting the world according to your purpose of life | Succeeding, playing |
| 90–80% | Emotional pain (denying and repressing the feelings) | Fighting, attacking |
| 66% **Neurotic** | Emotionally overwhelmed, psychic death (denying the purpose of life) | Fighting, defending |
| 50% | Escaping from here and now | Flight, running |
| 40% | Cannot escape, denying here and now | Freezing, helplessness |
| 33% **Psychotic** | Destructing the perception (wiping out, “blackness”, “closing eyes”, denying the mind) | Shocked, numb, lame |
| 20–10% **Hallucinating** (substituting perception) | Dreaming (perception and behavior not related to the outer world) | Dream state |
| 0% responsibility **Dead** | Unconscious, in coma (denying the body) | Physically dying, suicidal, evil and destructive |

## METHODS

The treatment of Anna and the principles behind it are described in the two first papers on Anna[16,17]. The case of Anna illustrates the inner logic and complexity of intensive holistic therapy, when it comes to be most difficult, where only a combination of intensive medical, psychiatric, and sexological treatment could set her free. The treatment was intensive existential holistic therapy with the theories of sexuality used to structure and interpret the elements and phases of the therapy strongly inspired by Freud, Jung, Reich, and Lowen. The focus on unconsciousness and the use of terms such as “projection” (transference) is an example of the (neo-)Freudian perspective.

The therapy started with addressing the layer of “quality of life-health-ability”; the next steps addressed the issues of love, consciousness, and sexuality and the third, final, and deepest layer of existential coherence. The patient ran though a series of steps in her personal process of metamorphosis (see Fig. 1) with three severe existential crises during the therapy:

- **A psychotic crisis** where the content of the stream of consciousness looked psychotic, while the patient was still with a part of her consciousness in present time, still able to perform normally, stayed in contact with the world and therefore not psychotic in the classical, psychiatric sense of the word. This was a necessary, but very painful phase of the therapy, where she integrated an old psychotic state of consciousness from her tormented childhood dominated by violence and sexual abuse.
- **A visionary crisis** where she understood her true nature as a human being and “remembered” the collective consciousness of mankind. In this phase, she “plugged” into being human again.
- **A suicidal crisis** where the content of the stream of consciousness looked like she wanted to die, while the patient also here with a part of her consciousness stayed in present time, still able to perform normally, to stay in contact with the outer world and therefore neither psychotic in the classical, psychiatric sense.

**TABLE 2. The Most Importance Sentences Anna Let Go of in Her Holistic Existential Therapy (as they appeared in the therapy)**

| | | | | | |
|---|---|---|---|---|---|
| 1. | I trouble other people | 40. | There is nothing to come after | 75. | I am worthless, am I not? |
| 2. | I am troublesome | | | 76. | I hate her |
| 3. | I am a burden | 41. | It is absurd | 77. | I loathe her |
| 4. | I am impossible | 42. | When I come, I am dying | 78. | She is nasty |
| 5. | It is my fault | 43. | I cannot stand having it inside me | 79. | She is disgusting |
| 6. | I am not good enough | | | 80. | I am a failure |
| 7. | I do not deserve to live | 44. | I cannot find my bearings at all | 81. | I am out in the space |
| 8. | I do not deserve life | | | 82. | I have failed |
| 9. | I have clocked out | 45. | This is the worst thing you have done to me | 83. | I cannot be in myself |
| 10. | This is unreal | | | 84. | I want to leave |
| 11. | I am empty | 46. | He is a pig | 85. | I am worthless |
| 12. | I am hollow | 47. | He disgusts me | 86. | Could he think of anything sexual? |
| 13. | I want to go away | 48. | He scares me | | |
| 14. | I want to live | 49. | He is like them | 87. | Nobody is there for me |
| 15. | I get relieved | 50. | I do not care | 88. | I am miserable |
| 16. | I decide – never mind | 51. | I bet you will get it! | 89. | Now it is enough! |
| 17. | She does not want to | 52. | He is cold | 90. | I cannot give birth to my child like that. – It will be a trauma for the baby |
| 18. | I do not want to hear it – ever | 53. | He is disgusting | | |
| | | | | 91. | He is going to kill me |
| | | 54. | He does not care | 92. | There is no room for me |
| 19. | I do not hear it | 55. | He is so violent | | |
| 20. | I do not want to know it | 56. | If you tell it to anybody, nobody will want to have you | | |
| 21. | I do not want to say it | 57. | I have got nobody | 93. | I am nothing |
| 22. | It is not my fault | | | 94. | She is going to kill me |
| 23. | This is not OK | | | 95. | It is really serious |
| 24. | This is my secret | 58. | It’s really bad | 96. | I feel sorry for myself |
| 25. | You should just know that you will be punished, harder then ever, if you tell it | 59. | I want to die | 97. | They are going to kill me |
| | | 60. | I cannot have this | 98. | I feel unwell |
| | | 61. | Unbearable pain | 99. | It is fine you came, and now you must leave |
| 26. | It is not me | 62. | Why didn’t they kill me? | 100. | I am good, warm and affectionate |
| 27. | I do not want to | 63. | You bet I will get it out | 101. | She is a schizophrenic |
| 28. | You get punished | 64. | I cannot do that | 102. | I am a schizophrenic |
| 29. | I do not want to hear it | 65. | I get smashed up | | |
| 30. | I can do nothing | 66. | I go to pieces | 103. | I am ashamed |
| 31. | I cannot stay anywhere | 67. | I will … | | |
| 32. | I bring warmth and joy | 68. | Be careful/do it carefully | 104. | I am very scared |
| 33. | I am ugly | 69. | It is not so bad | 105. | I am not important |
| 34. | I am afraid of men | 70. | I do not want any more | 106. | I bring life and joy |
| 35. | I feel sorry for myself | 71. | Nobody likes me | 107. | I bring life |

**Table 2, continued**

| | | |
|---|---|---|
| 36. She is too much | 72. It is odd | 108. What did happen? |
| 37. I am getting punished | 73. It is really curious | |
| 38. I do not need you | 74. I am not good, am I? | |
| 39. I need nobody | | |

*While letting go of these decisions, she healed her existence and recovered from a dysfunctional state caused by about the similar number of sexual abuse events in her childhood, including rape.

**TABLE 3**
**The Organization of the Sentences of Denial of Her Life in the Many Different Existential Dimensions[33] Fits to the Scheme of the Responsibility Scale and the Degeneration of Perception**

| 90% Responsibility | Emotional Pain (Denying and Repressing the Feelings) | Defending |
|---|---|---|
| 1. I have failed | 16. I am ugly | 24. I am worthless, am I not? |
| 2. Could he think of anything sexual? | 17. I am afraid of men | 25. I hate her |
| 3. I cannot give birth to my child like that. – It will be a trauma for the baby | 18. I feel sorry for myself | 26. I loathe her, |
| 4. I am good, warm and affectionate | 19. It is really bad | 27. She is disgusting |
| 5. I trouble other people<br>6. I am troublesome<br>7. I am a burden<br>8. It is my fault<br>9. I am not good enough<br>10. I do not deserve life<br>11. She does not want to<br>12. It is not my fault<br>13. This is not OK<br>14. I do not want to<br>15. I bring warmth and joy | 20. Unbearable pain<br>21. I do not want any more<br>22. It is not so bad<br>23. I am not good, am I? | 28. I am worthless<br>29. I am miserable<br>30. I feel so unwell<br>31. I am ashamed<br>32. I am very scared<br>33. I am not important |
| **50% Responsibility** | **Emotionally Overwhelmed, Psychic Death (Denying the Purpose of Life)** | **Fighting** |
| 34. I want to live<br>35. I get relieved<br>36. You get punished<br>37. She is too much<br>38. I am getting punished<br>39. When I come, I am dying | 40. I cannot stand having it inside me<br>41. I cannot find my bearings at all<br>42. He disgusts me<br>43. I do not care<br>44. I have got nobody<br>45. I want to die | 46. I want to go away<br>47. I cannot have this<br>48. Nobody likes me<br>49. I want to leave<br>50. Now it is enough!<br>51. It is really serious |

**Table 3, continued**

| **40% Responsibility** | **Escaping from Here and Now** | **Flight, Running** |
|---|---|---|
| 52. I have clocked out | 55. I don't hear it | 59. I can't stay anywhere |
| 53. I decide NEVER MIND | 56. I don't want to know it | 60. I am out in the space |
| 54. I do not want to hear it - ever | 57. I don't want to say it | 61. I cannot be in myself |
| | 58. I don't want to hear it | |
| **30% Responsibility** | **Cannot Escape, Denying Here and Now** | **Freezing, Helplessness** |
| 62. I am hollow | 68. He is a pig | 75. You bet I'll get it out |
| 63. It is not me | 69. He scares me | 76. I cannot do that |
| 64. I can do nothing | 70. He is cold | 77. I will … |
| 65. I do not need you | 71. He is disgusting | 78. Be careful/do it carefully |
| 66. I need nobody | 72. He does not care | 79. It is odd |
| 67. There is nothing to come after | 73. He is so violent | 80. It is really curious |
| | 74. If you tell it to anybody, nobody will want to have you | 81. I bring life and joy |
| | | 82. I bring life. |
| | | 83. What did happen |
| **20% Responsibility** | **Destructing the Perception (Wiping Out, "Blackness", "Closing Eyes", Denying the Mind)** | **Shocked, Numb, Lame** |
| 84. This is unreal | 88. He is like them | 89. I am a failure |
| 85. I am empty | | 90. There is no room for me |
| 86. This is a secret | | 91. I am nothing |
| 87. You should just know that you will be punished, harder than ever, if you tell it | | 92. It's fine you came, and now you must leave |
| **10% Responsibility** | **Hallucinating(Substituting Perception)** | **Dreaming (Perception and Behavior Not Related to the Outer World)** |
| 93. It is absurd | 94. She is a schizophrenic | 95. I am a schizophrenic |
| **0% Responsibility** | **Unconscious, in Coma (Denying the Body)** | **Physically Dying, Suicidal, Evil and Destructive** |
| 96. I do not deserve to live | 99. Why didn't they kill me? | 103. He is going to kill me |
| 97. This is the worst thing you have done to me | 100. I get smashed up | 104. They are going to kill me |
| | 101. I go to pieces | |
| 98. I bet you will get it! | 102. She is going to kill me | |

The intensity of her therapy followed a bell shaped curve (see Fig. 1) with a lot of minor arches rising and falling though the therapy. Interestingly "the tone or melody" of the processes changed during the process, from being dominated by painful emotions in the beginning, to understanding and revelation in the middle of the therapy and a focus on philosophy and “letting go” of negative beliefs in the end (see Fig. 2).

There are several methodological problems in using the Responsibility of Life Scale. First, the best way of operationalization has not yet been fully explored. Second, it would be necessary to know the inter-rater reliability of the scale before the scale could be meaningfully used in the clinical context. Interestingly, this pattern of “metamorphosis”, taking the patient from being like “the butterfly’s larvae” into the transformations state of pupae, finally into being the butterfly she was originally meant to be, seems to be so characteristic that the dominating quality of “feel-understand-let go” indicates where the patient is in the course of the therapy. This is very important as we often need a clue to find out, if there is more important, hidden material in the subconsciousness of the patient, so that therapy can be terminated. To create Table 2 and Table 3 was not so easy. Basically, we still need a systematic coding system to categorize the responses and we also need to prove that the responses have been coded in an objective manner. The presented meta-perspective of the therapy of Anna is therefore still a qualitative approach to understanding the process of intensive, holistic healing.

## DISCUSSION

The findings of these negative decisions and the content of these seem to be in agreement with the holistic process theory[18] and the holistic theory of mental illnesses[21,22]. The organization of the sentences according to the steps of the Responsibility for Life Scale was less successful, but still doable. The decay of existence seems to happen somewhat chaotically; the timeline of the appearance of the sentences in the therapy did not reveal much structure, as sentences with all kinds of content revealed themselves as disorderly and chaotic. It seemed that the destruction of life was done extremely creatively in every situation as a reduction, which then solves the problem in every case. It is very interesting that many different sentences can coexist and that the person has the resources to come back again and again, while still carrying the destructive sentences in her unconsciousness.

It seems fair to assume that the load of negative beliefs revealed by the therapy could have the effect of making Anna severely mentally ill, even schizophrenic, and that the integration of this material saved her mental health and general well being for life. When we experience life events with overwhelming emotional pain, we can escape this pain by making decisions in our mind that transfer responsibility from our existence to the surrounding world. By doing this, we destroy our being, our health, our quality of life, and our ability to function little by little. The case of Anna is an excellent example of such a systematic destruction of self, done to survive the extreme pressure on her existence from three men sexually abusing her systematically during many years of her childhood.

The most surprising aspect revealed by the study of Anna’s case is that the damage done to us by traumatic events is not on our body or our soul, but on our philosophy of life; the way we see and describe our world, life, our self, other people, and the world at large. The important consequence of this understanding is that we can heal our existence by letting go of the negative decisions taking in the painful and traumatic situations. By letting go of these life-denying sentences, we come back to life and to our natural responsibility for our own existence. We do not come back as a weak and wounded person; no, the real magic of life is that we seem to heal completely and in an absolute sense. We are able to wash all dirt from our bodies and minds, we are able to recover our character and our purpose of life, we are able to return to the brilliant state of being a free soul, and everything that happened, when fully integrated, will not affect us anymore.

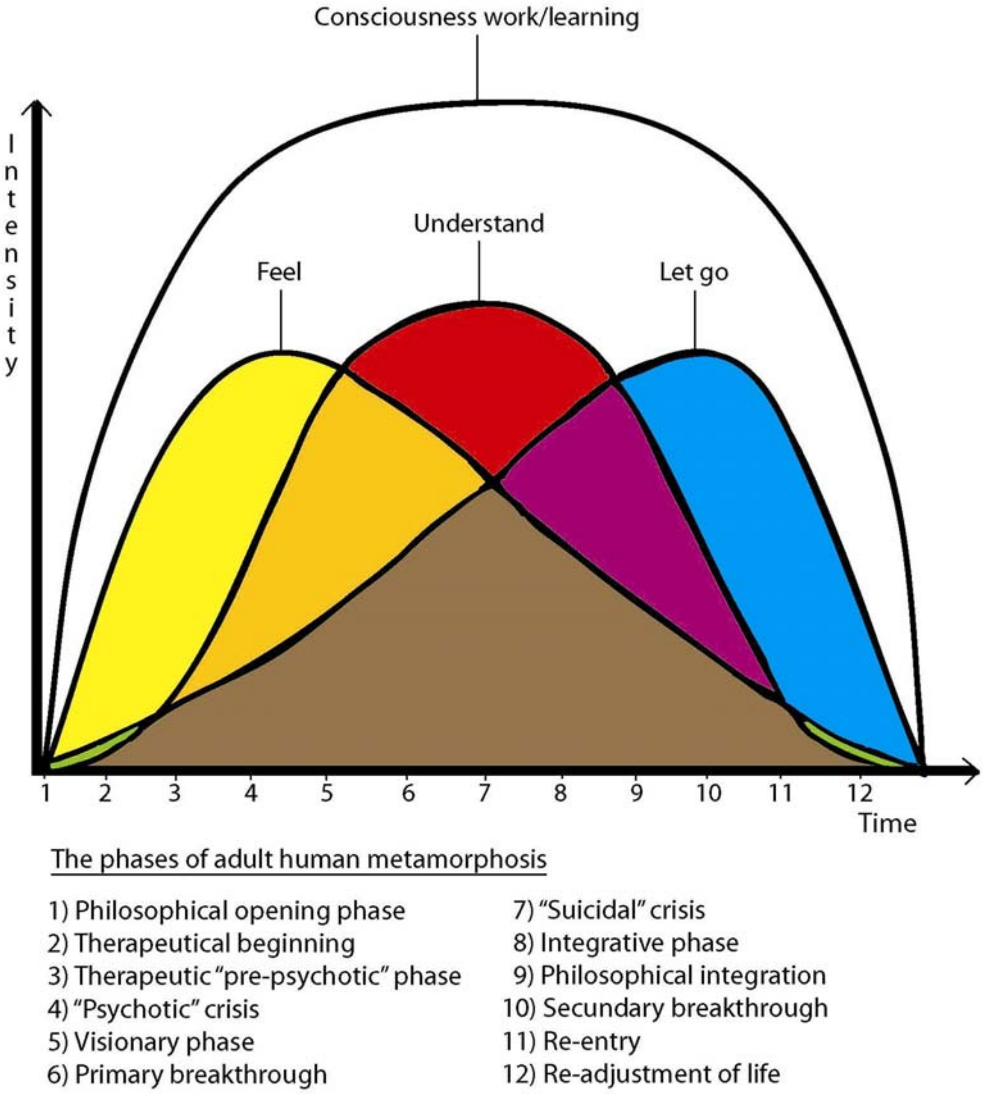

**FIGURE 1.** The process of holistic healing seen as three phases of feeling (yellow), understanding (red), and letting go (blue) of negative beliefs, attitudes, and decisions. As an end result, the process was improving the patient's philosophy of life and thus allowed the patient to rebalance existence and to assume responsibility for life. During the process, the patient's will re-established quality of life, health, and existential coherence, along with the ability to love, understand, and enjoy the whole spectrum of feelings and emotions, including sexuality. Many patients in intensive therapy experience the healing as a series of phenomena or breakthroughs and existential crises with characteristic content. The most intense crises are metaphorically called the "psychotic", the "visionary", and the "suicidal" crises. They include feelings of going insane, not knowing the world or oneself, and wanting to die. Knowing what is coming next in the course of therapy is of great help to the patient, making it much easier to confront and integrate the often extremely intense, painful emotions and states of being, arising from integrating the early childhood traumas. The 12 steps (see figure) are some possible steps in the process of healing and human transformation; understood though an ancient and powerful metaphor as the steps of "human metamorphosis".

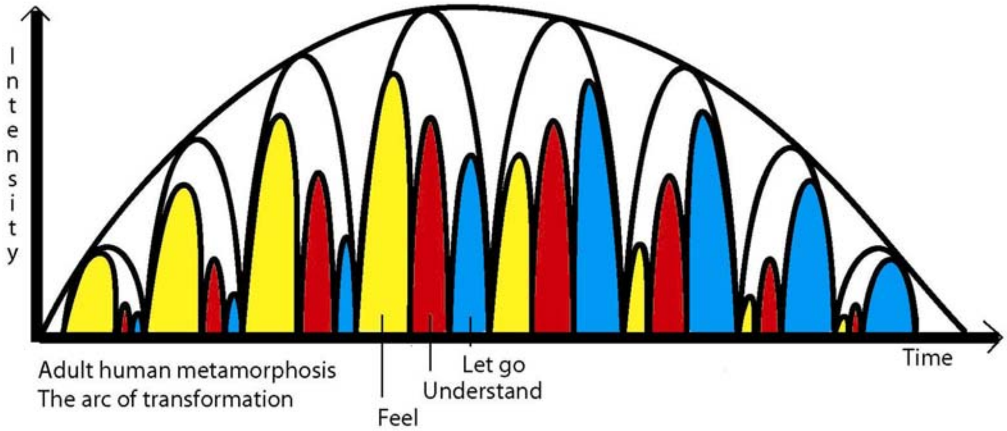

**FIGURE 2.** The arcs of transformation. The intensity of emotion, mental learning, and philosophical development follows a typical pattern in intensive holistic therapy. (We use the metaphor "adult human metamorphosis".)

The holistic healing of Anna's existence was done by existential holistic therapy. Although the processing did not always run smoothly, as she on several occasions projected very charged material on the therapists, the process ran all the way to full health and a good quality of life, thanks to her own will to recover completely. She wanted to be happy, she decided to take the process all the way to her personal happiness, and this was what made her keep working, until the day she could leave the clinic as a whole and renewed woman.

In our clinical experience, the advanced holistic medical toolbox has the tools needed for integrating even the most horrible of life events and traumas. The combination of holistic psychiatry, sexology, and rehabilitation was successful with even the most difficult and damaged of patients. Even when the patient was mentally ill and severely abused both violently and sexually during many years of her childhood, she could recover fully when she found love, trust, support, and holding enough to heal her existence and in this process, identify and let go of all her negative life decisions and systematically improve her philosophy of life. A "psychotic crisis" in the middle of the therapy seemed to be a good sign of healing and a "suicidal crisis" at the end of the therapy seemed to be a sign of the patient taking responsibility over her own life. It is important to underline that in spite of the dramatic metaphors of "psychosis" and "suicide", these metaphors address the content of her consciousness, not her general state of being; she was thus not psychotic in the classical psychiatric meaning of the word at any time during the therapy. After Anna, we have taken dozens of patients through similar processes without seeing any of them being endangered or harmed. This is very important, as this is the primary reason why even the most intensive, holistic existential therapy is completely safe for the patient, in spite of confronting the most horrible of feelings.

## ACKNOWLEDGMENTS

This study was supported by grants from IMK Almene Fond. The quality of life research was originally approved by the Copenhagen Scientific Ethical Committee under number (KF)V.100.2123/91.

## BIOSKETCHES

**Søren Ventegodt, MD,** is the director of the Nordic School of Holistic Health and Quality of Life Research Center in Copenhagen, Denmark. He is also responsible for a Clinical Research Clinic for Holistic Medicine in Copenhagen and is a popular speaker throughout Scandinavia. He has published numerous scientific or popular articles and a number of books on holistic medicine, quality of life, and quality of working life. His most important scientific contributions are the comprehensive SEQOL questionnaire, the very short QOL5 questionnaire, the integrated QOL theory, the holistic process theory, the life mission theory, and the ongoing Danish Quality of Life Research Survey, 1991–94 in cooperation with the University Hospital of Copenhagen and the late professor of pediatrics, Bengt Zachau-Christiansen, MD, PhD. E-mail: ventegodt@livskvalitet.org. Website: www.livskvalitet.org

**Birgitte Clausen, RN,** leader of a secluded ward for people with dementia. Teacher at Nordic School of Holistic Medicine. Dementia coordinator and consultant in Aarhus, Denmark with more than 20 years of experience in adult psychiatry and dementia. During the last 4 years, she has worked together with Søren Ventegodt on the "QOL as medicine project" and on developing existential holistic group therapy. Through her articles and lectures, she has disseminated "The New Culture of Dementia" in both Denmark and Japan. She has participated in the production of several videos to order to inspire relatives, medical staff, and volunteers to understand people with dementia. This project has been granted financial support from the Ministry of Social Affairs in Denmark. She has conducted the first Danish investigation on the effect of sufficient daylight on the human spirit, activity level, and biological rhythm. E-mail: villahvide@mail.dk

**Joav Merrick, MD, DMSc,** is professor of child health and human development affiliated with the Center for Multidisciplinary Research in Aging, Zusman Child Development Center, Division of Pediatrics and Community Health at the Ben Gurion University, Beer-Sheva, Israel; the medical director of the Division for Mental Retardation, Ministry of Social Affairs, Jerusalem; and the founder and director of the National Institute of Child Health and Human Development. Dr. Merrick has numerous publications in the field of child health and human development, rehabilitation, intellectual disability, disability, health, welfare, abuse, advocacy, quality of life, and prevention and received the Peter Sabroe Child Award for outstanding work on behalf of Danish Children in 1985 and the International LEGO-Prize ("The Children's Nobel Prize") for an extraordinary contribution towards improvement in child welfare and well being in 1987. E-mail: jmerrick@internet-zahav.net. Website: www.nichd-israel.com
